# Supplementary material for: Isothioureas, Ureas, and Their N-Methyl Amides from 2-Aminobenzothiazole and Chiral Amino Acids
Source: Molecules. 2019 Sep 18;24(18):3391. doi: 10.3390/molecules24183391 (PMC6767222; doi:10.3390/molecules24183391)
Supplement: Supplementary file 1 [file molecules-24-03391-s001.pdf]

molecules

MDPI

Article

Isothioureas, Ureas, and Their *N*-Methyl Amides from 2-Aminobenzothiazole and Chiral Amino Acids

Supplementry material

Table S1. Complementary data of SMe-isothiourea carboxylates **5a–f** and their methyl esters **8a–f**.

Compound	Yield (%)	Physical Appearance	M. P. (°C)	IR (cm^{-1})	$[\alpha]^{d}_{20}$ (g/mL)*	Elemental Analysis Found (Calculated)		
						C	H	N
**5a**.3H_2O	95	White powder	210 (dc)	3469<br>1643, 1548	-----	36.60<br>(36.97)	3.18<br>(2.80)	12.17<br>(16.76)
**5b**.2H_2O	81.4	Ionic liquid	-----	3488<br>1710, 1594	+77°<br>(1.87x10^{-2})	41.28<br>(40.79)	4.30<br>(3.39)	11.86<br>(11.90)
**5c**.1/2H_2O	86	White powder	250 (dc)	3480<br>1614, 1550	+194°<br>(2.64x10-2)	53.29<br>(52.57)	3.30<br>(3.61)	11.25<br>(10.82)
**5d**.3.5H_2O	62	White powder	------	3482<br>1745, 1592	-12°<br>(2.07x10^{-2})	47.04<br>(47.36)	4.80<br>(3.51)	9.08<br>(9.21)
**5e**.H_2O	67	Ionic liquid	------	3475<br>1738, 1560	------	48.69<br>(46.36)	4.63<br>(5.03)	12.17<br>(11.62)
**5f**.1.5H_2O	65	Ionic liquid	------	3486<br>1743, 1557	------	50.14<br>(47.32)	5.014<br>(5.49)	11.70<br>(10.95)
**8a**	83	White crystals	145-146	3474,<br>1745, 1551	-----	49.10<br>(48.81)	4.14<br>(4.40)	14.46<br>(14.23)
**8b**	80	White crystals	100-101	3446<br>1731, 1586	+147°<br>(1.93x10^{-2})	51.04<br>(50.32)	4.76<br>(4.83)	13.14<br>(13.54)
**8c**	75	White crystals	133-134	3470<br>1740, 1548	+260°<br>(1.42x10^{-2})	57.86<br>(58.22)	4.18<br>(4.58)	11.04<br>(11.32)
**8d**	56	White crystals	84-85	3466<br>1745, 1554	-34°<br>(2.73x10^{-2})	59.91<br>(59.22)	4.84<br>(4.93)	10.95<br>(10.91)
**8e**	49	Viscous liquid	----	3472<br>1738, 1560	-62°<br>2.06x10^{-2}	53.41<br>(53.18)	5.63<br>(5.72)	12.46<br>(12.08)
**8f**	51	Viscous liquid	----	3457, 1567	+183°<br>2.57x10^{-2}	54.70<br>(54.89)	5.98<br>(5.82)	11.96<br>(11.89)

*For compounds 5b–d, ethanol was used and for compounds 8b–d, chloroform was used.

Table S2. X-ray crystal data of compounds **8a**, **8b**, **8c**, and **13f**.

Compound	8a	8b	8c	13f
**Unit Cell**				
Cell axes [Å], a	8.0071(2)	7.4317(4)	37.7609(10)	9.1259(7)
b	22.7687(6)	8.5528(5)	8.3648(3)	7.1737(5)
c	8.3736(2)	12.1825(5)	5.7818(2)	14.8854(10)
Cell angles, α	90.000(0)	90.000(0)	90.000(0)	90.000(0)
β	115.327(1)	97.806(4)	90.000(0)	98.254(7)
γ	90.000(0)	90.000(0)	90.000(0)	90.000(0)
Crystal system	Monoclinic	Monoclinic	Orthorhombic	Monoclinic
Space group	$P\ 2_1/c$	$P\ 2_1$	$P\ 2_1\ 2_1\ 2$	$P\ 2_1$
Molecular Formula	$C_{12}H_{13}N_3O_2S_2$	$C_{12}H_{14}N_2S_2O_2$	$C_{18}H_{17}N_3O_2S_2$	$C_{17}H_{23}N_3O_2S_2$
Density (g cm^{-1})	1.42	1.34	1.35	1.26

Formula weight	295.4	309.4	371.5	365.5
No. Form. Units Z	4	2	4	2
**Reflection Data**				
No. Meas.	69781	12823	11370	34167
No. Uniq.	3699	3790	3698	5060
No. Obs.	3100	3099	3472	2485
**Current Refinement**				
No. Refln.	3699	3790	3698	5060
No. Param.	172	182	226	218
Delta-rho (eÅ^{-3}] max, min	0.290, -0.176	0.180, -0.133	0.197, -0.171	0.180, -0.231
R_all, R_obs	0.051, 0.039	0.050, 0.036	0.040, 0.036	0.137, 0.050
wR2_all, wR2_obs	0.111, 0.101	0.086, 0.078	0.100, 0.097	0.144, 0.110

Table S3. ^{1}H NMR data of compounds **5a–f** (DMSO-*d*$_6$).

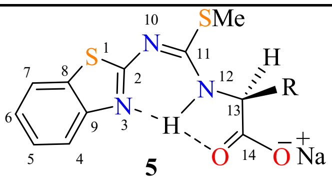

5

Comp.	H4	H5	H6	H7	N12H	SMe	C13H	R
**5a**	7.76<br>(d, 7.1)	7.32<br>(t, 7.6)	7.17<br>(t,7.2)	7.63<br>(d, 8.2)	10.66<br>(br)	2.44<br>(s)	3.62<br>(d, 3.8)	3.63<br>(d, 3.8)
**5b**	7.75<br>(d, 7.9)	7.31<br>(t, 7.6)	7.15<br>(t, 7.3)	7.63<br>(d, 8.0)	10.95<br>(d, 5.6)	2.42<br>(s)	3.91<br>(q, 7.6)	1.38<br>(d, 6.8)
**5c**	7.80<br>(d, 7.6)	no ob	no ob	7.63<br>(d, 7.9)	11.42<br>(d, 6.2)	2.31<br>(s)	4.78<br>(d, 6.2)	7.2-7.3<br>(m)
**5d**	7.76<br>(d, 7.9)	7.32<br>(t, 7.5)	no ob	7.54<br>(d, 7.9)	10.74<br>(d, 5.8)	2.36<br>(s)	4.12<br>(d, 3.8)	7.2, 3.14<br>(m, ddd, 13.6, 6.5,<br>5.0)
**5e**	7.77<br>(d, 7.0)	7.32<br>(d, 8.2,<br>7.0)	7.17<br>(d, 7.0,<br>7.6))	7.55<br>(d, 8.2)	10.87<br>(d, 8.8)	2.42<br>(s)	3.81<br>(dd, 4.1, 4.7,<br>3.5)	2.2, 0.9, 1.0<br>(m, d, d, 7.0)
**5f**	7.77<br>(d, 7.0)	7.31<br>(t, 7.6, 5.5)	7.17<br>8d, 7.6,<br>4.1)	7.57<br>(d, 8.2)	10.86<br>(d, 8.2)	2.35<br>(s)	3.90<br>(m)	1.66, 1.6, 0.92, 0.87<br>(m, m, d,d, 7.1, 6.4)

Table S4. ^{13}CNMR data of compounds **5a–f** (DMSO-*d*$_6$).

Comp.	C2	C4	C5	C6	C7	C8	C9	C11	SCH_3	C13	C14	R
**5a**	163.6	126.1	123.4	121.6	120.4	132.9	151.8	171.6	14.1	48.1	169.7	----
**5b**	163.2	126.1	123.5	121.5	120.6	131.8	151.7	174.0	14.1	55.1	171.6	20.7
**5c**	163.6	126.1	123.5	121.7	120.6	131.9	151.9	171.3	14.1	63.9	169.8	139.7
**5d**	164.4	126.1	123.5	121.6	120.4	131.6	151.6	172.5	14.0	61.2	171.3	56.5
**5e**	164.4	126.1	123.4	121.6	120.3	131.6	151.6	172.9	14.0	65.3	171.6	31.9, 19.9, 18.7
**5f**	163.8	126.0	123.3	121.5	120.3	131.5	151.5	173.7	13.9	58.7	171.5	43.3, 31.1, 25.1, 23.1

Table S5. ^{1}H NMR data of compounds **6a–f** (DMSO-*d*$_6$).

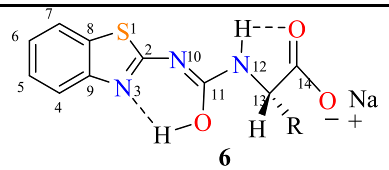

6

Comp.	H4	H5	H6	H7	OH	N12H	C13H	R
**6a**	7.80 (d, 7.1)	7.30 (d, 7.5)	7.60 (d, 7.5)	7.10 (d, 7.6)	no ob no ob	7.7 (t, 4.0)	3.6 (d, 4.0)	3.6 ----
**6b**	7.81 (d, 7.9)	7.31 (t, 7.3)	7.15 (t, 7.3)	7.59 (d, 7.9)	11.9 (br)	7.75 (d, 4.5)	3.94 (dq, 6.7)	1.29 (d, 6.7)
**6c**	7.87 (d, 7.6)	no ob	no ob	7.80 (d, 7.9)	11.85 (br)	8.3 (d, 6.2)	4.99 (d, 6.8)	7.1-7.4 (m)
**6d**	7.80 (d, 7.6)	7.30 (t, 7.0)	no ob	7.60 (d, 7.9)	12.0 (br)	8.0 (br)	4.25 (dd, 6.4)	2.98, 3.1, 7.2 (d, d, 13.1, 12.3, m)
**6e**	8.12 (d, 7.6)	7.63 (t, 7.0)	7.46 (t, 7.6)	7.94 (d, 7.6)	no ob	8.11 (d, (8.2)	4.37 (dd, 8.2)	2.48, 1.20, 1.22 (m, d, d, 7.1)
**6f**	7.80 (d, 7.6)	7.30 (t, 8.2)	7.13 (t, 7.6)	7.60 (d, 8.2)	12.3 (br)	7.90 (d, 8.2)	4.14 (m,br)	1.60, 1.50, 0.84, 0.86 (m, m, d, d, 6.4, 7.1)

Table S6. ^{13}CNMR data of compounds **6a–f** (DMSO-*d*$_6$).

Comp.	C2	C4	C5	C6	C7	C8	C9	C11	C13	C14	R
**6a**	157.8	119.0	121.1	121.5	125.5	132.0	150.1	175.1	45.1	164.4	----
**6b**	154.0	120.2	121.7	122.9	126.2	132.2	150.0	176.3	51.4	161.0	20.7
**6c**	153.4	120.0	121.5	122.7	126.5	131.9	149.7	172.5	60.0	160.5	143.1
**6d**	153.6	119.9	121.6	122.8	126.1	131.9	149.6	173.6	56.4	160.5	38.4, 126.0
**6e**	155.1	119.8	121.4	122.3	125.8	131.9	149.9	176.1	60.3	161.6	31.8, 20.2, 18.4
**6f**	154.3	119.9	121.4	122.5	125.9	131.9	149.8	177.3	53.9	160.9	43.6, 25.0, 23.7, 22.9

Table S7. ^{1}HNMR data of compounds **8a–d** ($CDCl_3$).

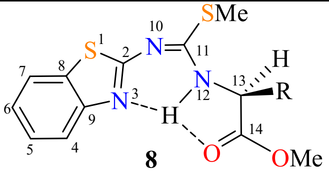

8

Comp.	H4	H5	H6	H7	N12H	SMe	C13H	R	OMe
**8a**	7.72 (d, 8.5)	7.34 (t, 7.9)	7.21 (t, 7.6)	7.69 (d, 9.3)	10.80 * (br)	2.55 (s)	4.23 (s)	4.23 (s)	3.83 (s)
**8b**	7.72 (d, 8.6)	7.34 (t, 8.4)	7.25 (t, 8.5)	7.69 (d, 8.5)	11.20 (br)	2.54 (s)	4.49 (q, 6.8)	1.61 (d, 7.0)	3.81 (s)
**8c**	7.80 (d, 7.8)	no ob	7.24 (t, 7.6)	7.72 (d, 7.6)	12.04 (br)	2.50 (s)	5.47 (br)	7.4-7.5 (m)	3.79 (s)
**8d**	7.68	no ob	no ob	7.66	11.23	2.36	4.70	3.28, 3.26, 7.2	3.75

	(d, 8.2)			(d, 9.2)	(br)	(s)	(br)	(s, s, m)	(s)
**8e**	7.74 (d, 4.9)	7.35 (t, 7.0)	7.21 (t, 7.6)	7.68 (d, 4.7)	11.41 (d, 8.2)	2.54 (dd,8.8,4.7)	4.30 (br)	2.37, 1.12, 1.09 (m, d, d, 7.0)	3.78 (s)
**8f**	7.70 (d,4.6)	7.35 (t, 8.2)	7.25 (t, 8.3)	7.68 (d, 4.1)	11.20 (br)	2.53 (s)	4.42 (br)	1.82, 1.83, 1.0, 0.98 (m, m, d, d, 6.8)	3.78 (s)

* Observed in DMSO-d_6 for **8a.**

Table S8. ^{13}C NMR data of compounds **8a–d** ($CDCl_3$).

Comp.	C2	C4	C5	C6	C7	C8	C9	C11	SCH3	C13	C14	R	OMe
**8a**	164.8	125.6	123.3	121.0	120.6	132.2	151.0	171.7	14.1	52.7	169.1	-	45.0
**8b**	164.0	125.6	123.3	121.0	120.7	132.1	151.0	171.7	14.0	52.3	172.3	19.3	52.8
**8c**	164.0	125.7	123.4	121.0	120.8	132.1	151.0	171.6	14.2	60.6	170.2	136.1	53.0
**8d**	164.2	125.6	123.3	121.0	120.6	132.2	151.0	171.5	14.2	58.2	171.1	39.1	52.6
**8e**	165.2	125.7	123.4	121.1	123.4	132.2	151.1	172.0	14.2	62.4	171.3	31.9, 19,3, 17.9	52.5
**8f**	164.6	125.6	123.3	121.0	120.5	132.2	151.0	172.2	14.0	55.4	171.8	42.0,25.0,22.7, 22.1	52.5

Table S9. ^{1}H NMR data of compounds **10a–f** ($CDCl_3$).

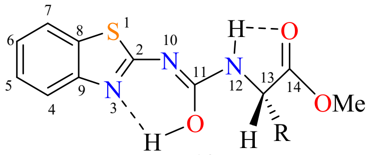

10

Comp.	H4	H5	H6	H7	OH	N12H	C13H	R	OMe
**10a**	7.75 (d, 8.1)	7.41 (t, 8,3)	7.27 (t, 8.2)	7.73 (d, 8.1)	11.0 no ob.	7.26 (d, 5.8)	4.2 (d, 5.5)	4.0 (d, 6.0)	3.70 (s)
**10b**	7.75 (d, 7.6)	7.38 (d, 8.0)	7.24 8d, 7.2)	7.70 (d, 7.6)	no ob.	No ob.	4.66 (q, 7.0)	1.54 (d, 7.1)	3.79 (s)
**10c**	7.88 (d, 7.6)	no ob	7.23 (d, 7.6)	7.65 (d, 7.6)	no ob.	no ob.	5.5 (s)	7.3-7.5 (m)	3.76 (s)
**10d**	7.71 (d, 8.2)	7.38 (d, 7.1)	no ob	7.65 d(10.5)	no ob	no ob	4.87 (q, 7.0)	7.15-7.25, 3.20 m, dd (5.8, 5.3)	3.73 (s)
**10e**	7.71 (d, 7.0)	7.34 (t, 7.0)	7.20 (t, 7.6)	7.69 d(7.0)	no ob	no ob	4.55 (dd, 4.7)	2.0, 0.79, 0.74 (m, d, d, 6.5)	3.74 (s)
**10f**	7.71 (d, 7.6)	7.35 (t, 7.6)	7.21 (t, 7.6)	7.68 (d, 7.6)	no ob	no ob	4.61 (m)	1.72, 1.67, 0.98, 0.96 (m, m, d, d, 6.5)	3.75 (s)

Table S10. ^{13}CNMR data of compounds **10a–f** ($CDCl_3$).

Comp.	C2	C4	C5	C6	C7	C8	C9	C11	C13	C14	R	OMe
**10a**	154.3	126.2	121.2	120.2	126.6	130.7	148.7	169.5	43.0	160.1	----	51.7
**10b**	154.2	126.1	121.2	120.1	123.5	130.9	148.8	173.6	49.1	161.4	18.5	52.6

**10c**	153.8	126.1	121.2	120.3	123.5	131.0	149.0	171.4	57.5	161.2	140, 129, 128, 127.1	57.5
**10d**	153.8	126.4	121.3	119.7	123.7	130.5	148.6	172.2	52.51	161.3	136, 129, 129, 127, 38	54.5
**10e**	154.2	125.6	120.8	119.9	122.8	131.4	149.2	172.6	57.9	160.5	30.95, 18.94, 17.51	51.9
**10f**	154.5	126.1	121.2	120.0	123.4	131.1	148.8	173.8	41.3	161.3	41.4, 24.9, 22.8, 21.9	52.4

Table S11. ^{1}H NMR data of compounds **11a–f** ($CDCl_3$).

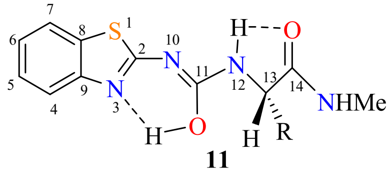

11

Comp.	H4	H5	H6	H7	OH	N12H	C13H	N14H	R	NMe
**11a**	7.88 (d, 8.3)	7.36 (t, 7.1)	7.21 (t, 7.6)	7.63 (d, 8.2)	10.8 (br)	7.21 (t, 5.2)	3.79 (d, 5.3)	7.96 (q, 4.1)	3.79 (d, 5.3)	2.62 (d, 4.7)
**11b**	7.86 (d, 7.5)	7.36 (t, 7.1)	7.21 (t, 7.5)	7.62 (d, 8.0)	no ob.	7.36 (t, 6.3)	4.25 (q, 7.0)	8.1 (q, 4.6)	1.27 (d, 7.0)	2.61 (d, 4.6)
**11c**	7.84 (d, 8.2)	no ob	7.15 (d, 7.6)	7.61 (d, 7.6)	no ob	7.9 (d, 7.0)	5.37 (d, 7.7))	8.4 (q, 7)	7.2-7.5 (m)	2.61 (d, 4.1)
**11d**	7.86 (d, 7.7)	7.36 (t, 7.1)	no ob	7.62 (d, 8.0)	10.6 (br)	7.10 (d, 7.9)	4.48 (q, 8.1)	8.17 (q, 4.5)	7.1-7.3(m) 2.95 (ddd, 21.7, 13.7, 6.7)	2.61 (d, 4.5)
**11e**	7.86 (d, 8.2)	7.35 (t, 7.6)	7.20 (t, 7.6)	7.62 (d, 7.7)	10.6 (br)	7.06 (d, 8.7)	4.14 (dd, 8.7, 5.9)	8.10 (q, 4.6)	1.98, 0.87, 0.83 m, d, d (7.1)	2.6 (d, 4.6)
**11f**	7.85 (d, 8.3)	7.33 (t, 7.2)	7.18 (t, 7.6)	7.61 (d, 7.9)	10.5 (br)	7.0 (d, 8.2)	4.22 (m)	8.13 (q, 4.7)	1.55, 1.46, 0.87, 0.85 m, m, d, d (2.4)	2.57 (d, 4.7)

Table S12. ^{13}C NMR data of compounds **11a–f** ($CDCl_3$).

Comp.	C2	C4	C5	C6	C7	C8	C9	C11	C13	C14	R	NMe
**11a**	154.3	126.2	123.1	121.8	120.1	131.8	159.5	169.5	43.0	160.1	---	25.9
**11b**	153.6	126.3	123.2	121.7	120.1	131.8	149.4	173.0	49.3	160.1	19.8	26.0
**11c**	153.8	126.1	123.0	121.7	120.1	131.8	149.5	170.5	57.1	160.3	140	26.1
**11d**	153.6	126.3	123.2	121.8	120.2	131.8	149.5	171.6	54.8	160.0	138, 130, 129, 127, 39	26.0
**11e**	154.0	126.2	123.1	121.8	120.2	131.8	149.5	171.6	63.3	160.0	30.4, 17.8,17.3	25.5
**11f**	153.9	126.2	123.0	121.7	120.1	131.9	149.5	172.7	52.1	160.1	42.4, 24.8, 23.3, 22.3	26.0
